# MUSA: A Multimodal MRI-Based Major Depressive Disorder Classification Model with a Unified ROI Semantic Space

**DOI:** 10.3390/s26206555

**Published:** 2026-10-15

**Authors:** Honghua Wang, Xiuwei Peng, Han Wu, Jinbao Xiang, Jingyu Liu, Yunhong Ding, Lin Wu, Bo Qi

**Affiliations:** 1 School of Computer Science and Information Engineering, Harbin Normal University, Harbin 150025, China; 2023030238@stu.hrbnu.edu.cn (H.W.); 2023021215@stu.hrbnu.edu.cn (X.P.); 2024030103@stu.hrbnu.edu.cn (L.W.); 2 School of Computer Science and Internet of Things, Chongqing Institute of Engineering, Chongqing 400056, China; 05150@cqie.edu.cn (H.W.); xiangjinbao@stu.cqie.edu.cn (J.X.); 3 School of Astronautics, Harbin Institute of Technology, Harbin 150001, China; 22b918100@stu.hit.edu.cn

**Keywords:** major depressive disorder, multimodal MRI, multimodal fusion, brain parcellation, region of interest

## Abstract

Major depressive disorder (MDD) is a common disabling psychiatric disorder mainly diagnosed through clinical interviews and rating-scale assessments. Magnetic resonance imaging (MRI) provides a promising basis for computer-aided MDD identification, particularly through multimodal models that can integrate structural and functional brain information. However, existing multimodal MRI fusion models often use data-level fusion or post hoc feature concatenation, potentially overlooking differences in spatial resolution, voxel organization, and region of interest (ROI)-level semantic correspondence. To address this issue, this study proposes MUSA, a unified ROI semantic space model for multimodal MRI-based MDD classification. An Automated Anatomical Labeling (AAL) atlas-guided ROI semantic space establishes consistent brain-region correspondence across heterogeneous MRI features. An ROI-level 3D Vision Transformer (3D ViT) encodes ROI voxel information into fixed-dimensional representations, allowing structural, local functional, and connectivity information to be organized and integrated within a consistent brain-region semantic space. Experiments on 2380 participants from REST-meta-MDD validated MUSA for MDD and normal controls (NCs) classification. The feature combination of white matter (WM), functional connectivity (FC), regional homogeneity (ReHo), and degree centrality (DC) achieved 79.20% ± 0.65% accuracy and an AUC of 87.08% ± 0.51%. Brain-region importance analysis identified classifier-derived discriminative patterns across frontoparietal, sensorimotor, visual, temporal, and limbic-related regions.

## 1. Introduction

Major depressive disorder (MDD) is one of the psychiatric disorders with the greatest global disease burden and is characterized by a high prevalence, a high recurrence rate, and a high risk of disability [1]. The clinical diagnosis of MDD currently relies mainly on symptomatology-based criteria, psychiatric interviews, and rating-scale assessments [2]. However, these assessments are partly subjective and may be affected by symptom heterogeneity and individual differences. Objective neuroimaging evidence that can support auxiliary diagnosis remains limited [3]. Although machine learning models have shown potential for assisting MDD identification, they still face clear challenges in individual-level discrimination and clinical translation [4]. Therefore, exploring MRI-based auxiliary classification models using objective neuroimaging data remains of important research significance.

With the development of neuroimaging and computational psychiatry, magnetic resonance imaging (MRI) has become an important data source for revealing MDD-related brain abnormalities and constructing auxiliary diagnostic models. MDD is not restricted to a single brain region or pathway. Instead, it involves heterogeneous alterations in brain structure, local functional activity, and interregional connectivity [5,6]. Structural MRI (sMRI) can characterize morphological differences in brain tissue, whereas resting-state fMRI (R-fMRI) can reflect spontaneous functional activity and brain network interactions. Local functional metrics derived from R-fMRI, such as amplitude of low-frequency fluctuation (ALFF), regional homogeneity (ReHo), degree centrality (DC), and voxel-mirrored homotopic connectivity (VMHC), provide information about regional functional abnormalities [7,8,9,10]. Functional connectivity (FC) further describes interaction patterns among brain regions and can reflect MDD-related network dysfunction [11]. However, the individual-level diagnostic performance of single imaging representations remains limited by disease heterogeneity [12]. Therefore, structural features, local functional features, and connectivity features may provide complementary information for MDD classification [13,14].

Motivated by this, multimodal modeling that integrates sMRI features, local functional metrics derived from R-fMRI, and FC features provides an important direction for improving MDD classification. However, when heterogeneous MRI features are extracted independently and combined mainly at the data or feature level, explicit ROI-level correspondence across feature types may be weakened [15,16,17,18]. This strategy may weaken the correspondence among brain regions across features, especially when different features differ in spatial resolution, voxel organization, and biological meaning [19]. In addition, voxel-level representations preserve local information but usually contain high dimensionality and redundancy, whereas region of interest (ROI)-level representations are more structured and interpretable but may lose fine-grained intra-ROI information. Therefore, an effective multimodal MRI model should establish consistent ROI-level correspondence across features while preserving discriminative voxel information within each ROI.

To address these issues, this study proposes MUSA, a unified ROI semantic space model for multimodal MRI-based MDD classification. The core idea of MUSA is to establish a consistent brain-region semantic space for heterogeneous MRI features and organize multimodal information according to explicit ROI correspondence before downstream integration and classification. Specifically, the Automated Anatomical Labeling (AAL) atlas [20] is used to guide spatial organization and establish consistent ROI correspondence across sMRI features and R-fMRI-derived local functional metric maps. Within this unified ROI semantic space, imaging-derived ROI voxel information is encoded into fixed-dimensional ROI representations using an ROI-level 3D Vision Transformer (3D ViT)-based encoder [21]. FC is further organized as ROI-level connectivity fingerprints and integrated with imaging-derived ROI representations at the same brain-region semantic level. In this way, MUSA jointly models structural, local functional, and connectivity information while maintaining brain-region-level interpretability for classification between patients with MDD and normal controls (NCs).

The main contributions of this study are summarized as follows:

1. This study presents MUSA, a multimodal MRI-based model for MDD classification. The proposed model establishes a unified ROI semantic space to organize structural, local functional, and functional connectivity information according to consistent brain-region correspondence across heterogeneous MRI features with different spatial resolutions, voxel organizations, and feature forms.
2. This study constructs an ROI-level multimodal representation strategy. This representation strategy organizes different MRI features at the brain-region level and preserves intra-ROI voxel information, thereby providing a consistent semantic basis for the joint modeling of structural features, local functional metrics, and FC fingerprints.
3. This study validates the effectiveness of the proposed model on the large-scale REST-meta-MDD dataset and systematically compares the effects of single features, within-modality combinations, and multimodal combinations on MDD/NCs classification performance.

## 2. Related Work

In recent years, MRI-based MDD identification has received sustained attention. With the continued application of large-scale open datasets such as REST-meta-MDD, researchers have been able to investigate MDD-related structural abnormalities, functional abnormalities, and disease heterogeneity using larger samples. This has promoted the shift of MRI-based MDD classification studies from small-sample validation toward more reliable model evaluation and method comparison [22,23]. Existing studies have mainly focused on structural information from sMRI, local functional information derived from R-fMRI, and interregional connectivity information represented by FC. To present representative studies conducted on the REST-meta-MDD dataset in recent years, this study summarizes related work in Table 1.

### 2.1. ROI-Level Statistical Feature-Based Models for MDD Classification

Many conventional MRI-based MDD classification studies mainly relied on feature engineering. Low-order statistical features or functional connectivity features were usually extracted based on predefined brain regions, such as ROI-level statistical summaries and interregional functional connectivity strength [11,24,25]. These handcrafted features were then fed into shallow classifiers, such as support vector machines, logistic regression, random forests, or gradient boosting classifiers. These models are easy to implement and have relatively low computational cost. They also provide relatively direct interpretability because the extracted features are usually associated with predefined brain regions or neuroimaging measurements. However, their performance is often limited by the representation ability of handcrafted features. Low-order ROI-level statistical features or functional connectivity-based statistical features may be insufficient to characterize complex MDD-related discriminative patterns. In particular, ROI-level statistical descriptors usually summarize each brain region using low-order values, which may not sufficiently preserve fine-grained voxel information within each ROI.

### 2.2. Single-Modality MRI-Based Models for MDD Classification

Existing single-modality image-based models usually use whole-brain sMRI volumes or R-fMRI-derived local functional metric maps as inputs. These data are commonly modeled using three-dimensional convolutional neural networks (3D CNNs), lightweight vision backbones, or Transformer-based models to learn MDD-related structural or functional abnormality patterns [26,27]. Whole-brain input strategies can preserve global spatial information and allow deep models to learn distributed imaging patterns [28]. However, the learned features are usually not explicitly organized according to predefined anatomical brain regions. As a result, the correspondence between model representations and specific ROIs is not direct, and brain-region-level interpretation often relies on post hoc visualization or attribution analysis.

### 2.3. Multimodal MRI Fusion Models for MDD Classification

Because a single imaging representation is usually difficult to fully characterize the complex neural mechanisms of MDD, multimodal MRI fusion has gradually become an important research direction. Existing models integrate sMRI and R-fMRI information through adaptive multimodal feature integration, evolutionary neural architecture search, and multimodal attention or dynamic disentanglement mechanisms [16,29,30]. Multi-stream 3D CNN models, such as Multi-stream 3D DenseNet121, have also been used to jointly model structural and functional MRI features [31]. These findings suggest that structural and functional information can reflect MDD-related abnormalities at different levels, and their joint modeling can provide a more comprehensive disease representation than using a single imaging source.

**Table 1:** MDD classification studies using the REST-meta-MDD dataset.

Author	Modality	Method	MDD/NCs	Accuracy (ACC, %)
Shi et al. [24]	R-fMRI	XGBoost	1021/1100	72.80
Wang et al. [30]	sMRI + R-fMRI	AMNI	282/251	65.00
Gallo et al. [11]	R-fMRI	SVM/GCN	1255/1083	61.00
Dai et al. [27]	R-fMRI	Transformer-Encoder	832/779	68.61
Li et al. [16]	sMRI + R-fMRI	M-ENAS	507/492	73.68
Wei and Gao [31]	sMRI + R-fMRI	Multi-stream 3D DenseNet121	830/771	69.38
Chen et al. [29]	sMRI + R-fMRI	MMDD	1300/1128	77.76
Cong et al. [26]	sMRI	3D RepViT	1186/1040	74.89

Recent studies have further explored multisite robustness, domain generalization, site-invariant modeling, graph-based learning, and uncertainty-aware multimodal fusion for MDD classification [32,33,34]. More broadly, recent reviews of AI-enabled biomedical signal analysis and deep learning-based medical image segmentation further illustrate the rapid development of representation learning and intelligent analysis methods for complex biomedical data [35,36]. In parallel, interpretable depression assessment has expanded beyond neuroimaging-only settings; for example, DEPART investigates interpretable depression assessment using in-the-wild video data [37]. These developments suggest that the ROI-level representation in MUSA could potentially serve as an imaging branch in broader multimodal frameworks incorporating behavioral, speech, video, or clinical information.

However, many existing multimodal MRI fusion models still combine whole-brain image features, ROI-level statistical features, FC network representations, or independently extracted branch features after feature extraction. In these cases, multimodal fusion is mainly performed at the sample level or feature level, and explicit ROI-level semantic correspondence across structural, local functional, and connectivity information may not be fully preserved. This issue becomes more evident when sMRI features, R-fMRI-derived local functional metric maps, and FC features differ in spatial resolution, voxel organization, and feature form. Accordingly, MUSA focuses on organizing heterogeneous MRI-derived information within a unified ROI semantic space before downstream integration and classification, rather than introducing a fundamentally new multimodal fusion mechanism.

Overall, ROI-level statistical feature-based models, single-modality whole-brain image-based models, and multimodal MRI fusion models have all contributed to MDD classification. However, several limitations remain in the current multimodal MRI setting. First, low-order ROI-level statistical descriptors may not sufficiently preserve fine-grained voxel information within each ROI. Second, whole-brain image-based models usually learn distributed imaging patterns without explicit organization according to anatomical ROIs, which may limit direct brain-region-level interpretation. Third, many multimodal fusion models integrate independently extracted features at the sample or feature level, and explicit ROI-level semantic correspondence across structural, local functional, and connectivity information may not be fully preserved. To address these limitations, this study focuses on unified ROI semantic space organization, ROI-level voxel encoding, and multimodal joint modeling, which are described in detail in Section 3.

## 3. Materials and Methods

### 3.1. REST-Meta-MDD Dataset

This study used publicly available MRI data from the REST-meta-MDD Consortium (approval number U0485). The data can be downloaded from the Psychological Science Data Bank at https://rfmri.org/REST-meta-MDD (accessed on 28 September 2026). This dataset is one of the largest neuroimaging resources currently available in depression research. It includes sMRI data, R-fMRI data, and clinical phenotypic information from 2428 participants (1300 patients with MDD and 1128 NCs) [38]. According to the REST-meta-MDD Consortium, the dataset contains 48 duplicate samples. Therefore, 2380 participants were finally included in this study, including 1276 patients with MDD and 1104 NCs. The demographic information of the included participants is shown in Table 2.

### 3.2. Data Preprocessing

sMRI and R-fMRI data were preprocessed using the MATLAB-based standard preprocessing pipeline of Data Processing Assistant for Resting-State fMRI (DPARSF) [39]. For functional imaging data, ALFF was selected from the results processed with Slice Timing Correction, Realign, Covariates Removed (without global signal regression), and Spatial Normalization. On this basis, ReHo, DC, and ROI time series were selected from the data further processed with an additional Filter step (0.01–0.1 Hz). On this basis, VMHC was selected from the data further processed with the additional steps of Normalized to a symmetric template and Smooth. For structural imaging data, gray matter (GM), white matter (WM), and cerebrospinal fluid (CSF) volume density maps in Montreal Neurological Institute (MNI) space were selected after voxel-based morphometry (VBM) processing in Statistical Parametric Mapping (SPM). The fixed sizes of the local functional metric maps derived from R-fMRI and sMRI volumetric data used in this study were 61×73×61 and 121×145×121, respectively.

These relatively standardized preprocessing procedures were consistently applied to the imaging data used in this study and may help reduce variability related to image format, spatial normalization, and preprocessing operations across subjects. However, they do not explicitly eliminate site-related distribution differences arising from scanner hardware, acquisition protocols, imaging parameters, or participant characteristics.

**Figure 1:**
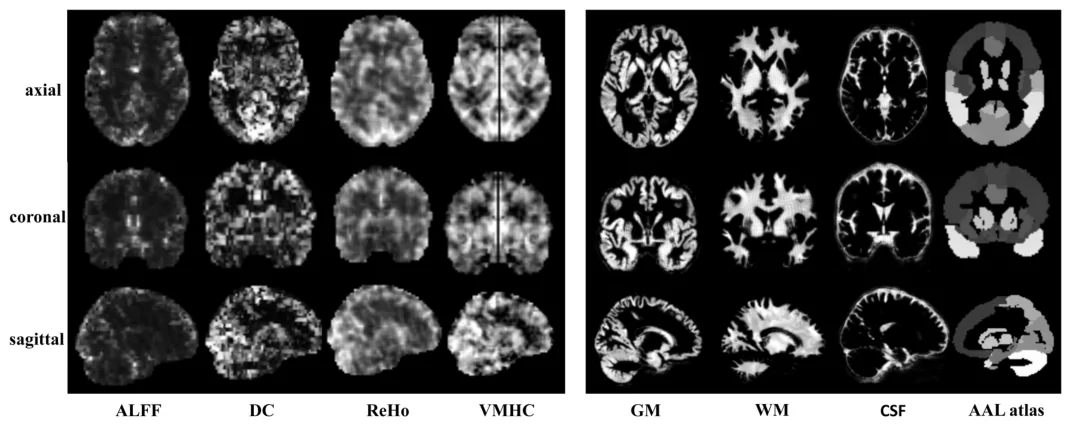
Example slices of MRI features and the AAL atlas at 18 mm.

Examples of the corresponding MRI features and the AAL atlas at the same slice location are shown in Figure 1.

**Figure 2:**
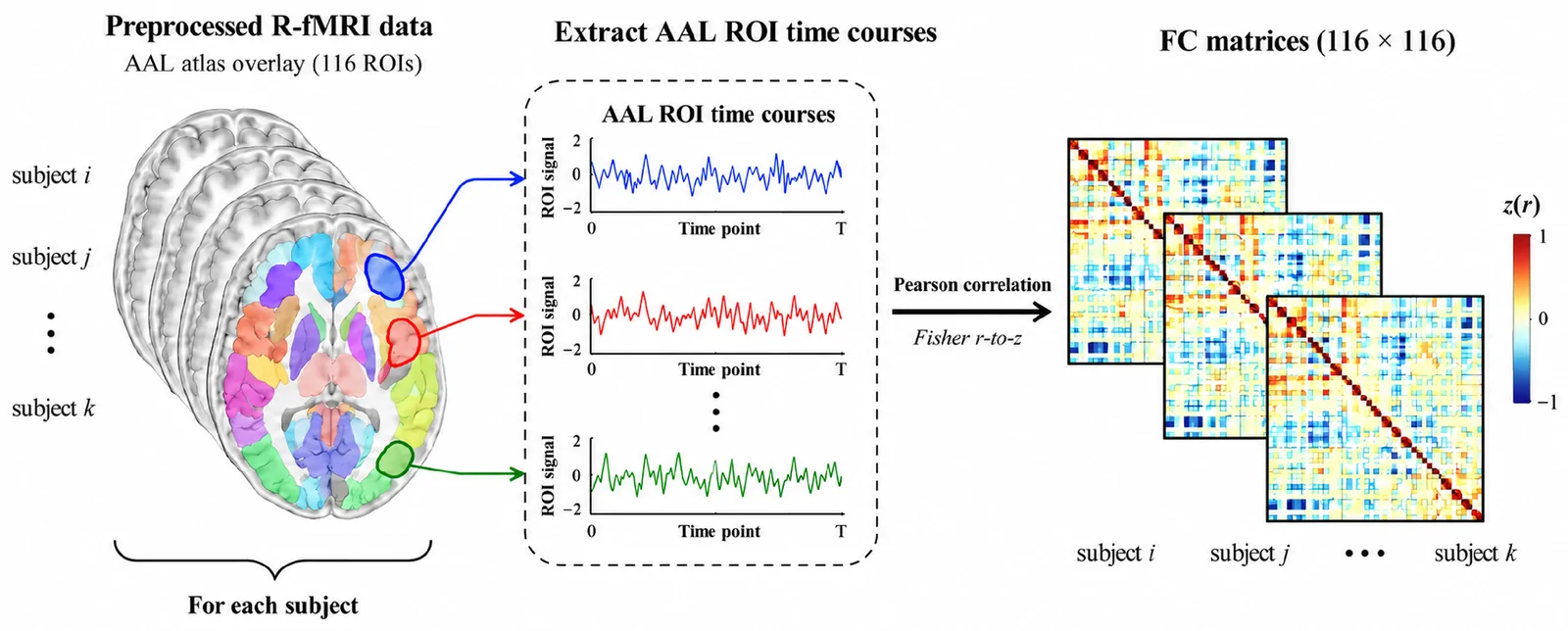
Construction of FC matrices from AAL atlas-defined ROI time courses.

In FC construction, this study used ROI time courses extracted based on the AAL atlas to characterize interregional functional connectivity. Specifically, the AAL atlas contains 116 ROIs. As shown in Figure 2, for each subject, the AAL atlas was overlaid on the preprocessed R-fMRI data to define 116 ROIs and extract the corresponding ROI time courses. Each ROI time course represents the average signal change of the corresponding brain region during R-fMRI scanning. Subsequently, Pearson correlation coefficients were calculated for all ROI pairs, followed by Fisher r-to-z transformation to obtain a 116×116 FC matrix for each subject.

For the *i*-th subject, let $s_{i,p}$ and $s_{i,q}$ denote the time courses of the *p*-th and *q*-th ROIs, respectively. The Pearson correlation coefficient between these two ROI time courses was calculated as:

(1) $$r_{i,pq}=\frac{\sum_{t=1}^{T_{i}}\left(s_{i,p}\left(t\right)-{\bar{s}}_{i,p}\right)\left(s_{i,q}\left(t\right)-{\bar{s}}_{i,q}\right)}{\sqrt{\sum_{t=1}^{T_{i}}{\left(s_{i,p}\left(t\right)-{\bar{s}}_{i,p}\right)}^{2}}\sqrt{\sum_{t=1}^{T_{i}}{\left(s_{i,q}\left(t\right)-{\bar{s}}_{i,q}\right)}^{2}}}$$

where $T_{i}$ denotes the number of time points of the *i*-th subject, and ${\bar{s}}_{i,p}$ and ${\bar{s}}_{i,q}$ denote the mean values of the corresponding ROI time courses.

Subsequently, Fisher r-to-z transformation was applied to the Pearson correlation coefficient:

(2) $$z_{i,pq}=\frac{1}{2}\ln\left(\frac{1+r_{i,pq}}{1-r_{i,pq}}\right)$$

The above calculation was repeated for all ROI pairs. Through this process, each subject was represented by an ROI × ROI FC matrix. Since the AAL atlas contains 116 ROIs, each FC matrix had a size of 116×116. Each row of the matrix represents the connectivity pattern between one ROI and all other ROIs. Therefore, the FC matrix was used as ROI-level connectivity fingerprints for multimodal fusion in the subsequent MUSA model.

### 3.3. MUSA

**Figure 3:**
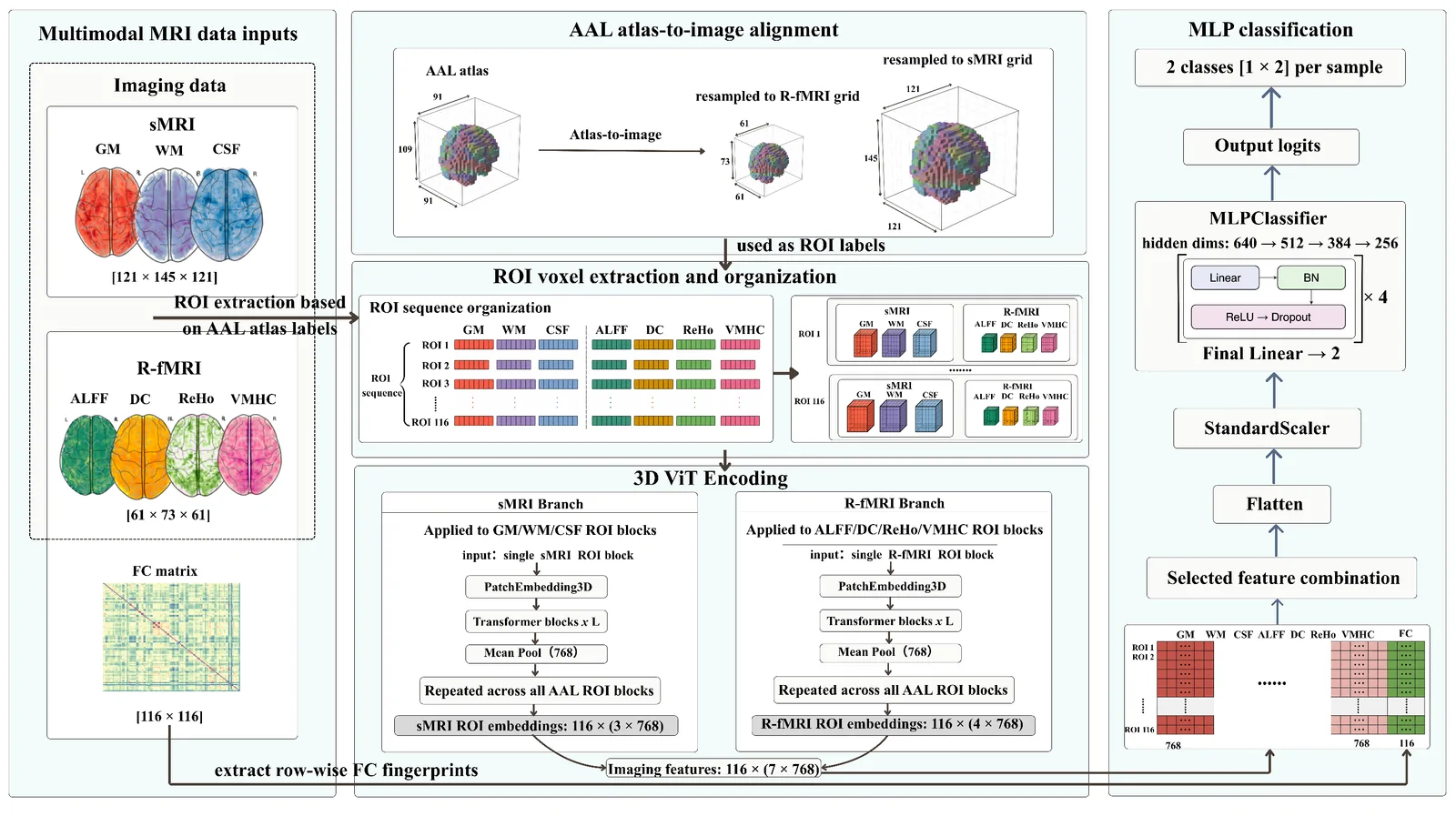
Overall model structure of MUSA, including multimodal MRI inputs, unified ROI semantic organization, ROI-level feature encoding, multimodal feature integration, and MLP classification.

In this study, MUSA is designed to organize multimodal MRI features within a unified ROI semantic space for MDD/NCs classification. Within this space, each ROI voxel sequence is reorganized into a three-dimensional local block and mapped into a fixed-dimensional ROI representation using a 3D ViT-based ROI encoder. These ROI representations are then organized together with ROI-level FC fingerprints at the same brain-region semantic level for subsequent integration and classification. The overall model structure of MUSA is shown in Figure 3.

As shown in Figure 3, MUSA mainly consists of three sequential steps. Step 1 is multimodal MRI data input. The model takes the preprocessing outputs described in Section 3.2 as inputs, including sMRI feature maps, R-fMRI-derived local functional metric maps, and FC matrices. The sMRI features include GM, WM, and CSF, while the R-fMRI-derived local functional metrics include ALFF, DC, ReHo, and VMHC. Step 2 is unified ROI semantic organization and ROI-level encoding. First, the AAL atlas is resampled into the image space of each imaging feature to establish ROI correspondence across different spatial resolutions. Based on the aligned ROI labels, voxel sequences are extracted from each ROI and reorganized into ROI-level three-dimensional blocks. These ROI blocks are then encoded by the 3D ViT-based ROI encoder to obtain fixed-dimensional ROI representations. Step 3 is ROI-level feature integration and classification. According to the selected feature combination, each row of the FC matrix is treated as the connectivity fingerprint of the corresponding ROI. Then, imaging-derived ROI representations and ROI-level FC fingerprints are concatenated along the feature dimension. The fused ROI-level feature matrix is flattened and standardized before being input into the multilayer perceptron (MLP) classifier, which outputs the predicted class probabilities for MDD/NCs classification. The overall computational procedure of MUSA is summarized in Algorithm A1 in the Appendix A.

#### 3.3.1. ROI Spatial Alignment and Voxel Extraction

The purpose of this subsection is to convert the sMRI feature maps and R-fMRI-derived local functional metric maps obtained in Section 3.2 into ROI-level voxel sequences. These sequences provide the inputs for ROI block construction and 3D ViT-based feature encoding in Section 3.3.2. The AAL atlas divides the whole brain into R=116 predefined ROIs. Because sMRI feature maps and R-fMRI-derived local functional metric maps differ in spatial resolution and voxel grids, this study adopts an atlas-to-image strategy to resample the AAL atlas into the image space of each imaging feature. Nearest-neighbor interpolation is used to preserve discrete ROI labels. After ROI spatial alignment and voxel extraction, each imaging feature is organized into 116 ROI voxel sequences. In this way, heterogeneous MRI features can be organized into a unified ROI semantic space, thereby establishing consistent brain-region correspondence across different modalities and features.

For the *i*-th subject under modality *m*, the target image is denoted as $Y_{i}^{m}$. Here, *m* denotes a specific imaging feature, such as GM, WM, CSF, ALFF, DC, ReHo, or VMHC. Let $A_{\mathrm{atlas}}$ denote the affine matrix of the AAL atlas, and let $A_{\mathrm{img},i}^{m}$ denote the affine matrix of the target image $Y_{i}^{m}$. First, the mapping matrix from the target-image voxel coordinates to the AAL atlas voxel coordinates is calculated as:

(3) $$T_{i}^{m}=A_{\mathrm{atlas}}^{-1}A_{\mathrm{img},i}^{m}$$

where $T_{i}^{m}$ denotes the coordinate mapping from the target image space to the atlas space for the *i*-th subject under modality *m*. For any voxel position *p* in the target image space, its homogeneous coordinate is denoted as p. The corresponding coordinate in the AAL atlas space is calculated as:

(4) $$p_{\mathrm{atlas}}=T_{i}^{m}p$$

Based on this mapping, the AAL atlas is resampled into the image space of the target image $Y_{i}^{m}$, producing the aligned ROI label map $L_{i}^{m}$. Because the ROI labels in the AAL atlas are discrete integer labels, nearest-neighbor interpolation is used to avoid label mixing. In the aligned ROI label map, $L_{i}^{m}\left(p\right)=r$ indicates that voxel position *p* in the target image belongs to the *r*-th ROI, and $L_{i}^{m}\left(p\right)=0$ indicates the background.

Based on the aligned ROI label map $L_{i}^{m}$, the voxel index set of the *r*-th ROI under modality *m* is defined as:

(5) $$\Omega_{i,r}^{m}=\left\{p|L_{i}^{m}\left(p\right)=r\right\}$$

where $\Omega_{i,r}^{m}$ denotes all voxel positions belonging to the *r*-th ROI for the *i*-th subject under modality *m*. Then, the voxel values at these positions are extracted from the target image $Y_{i}^{m}$, yielding the voxel sequence of the *r*-th ROI:

(6) $$x_{i,r}^{m}=\left\{Y_{i}^{m}\left(p\right)|p\in \Omega_{i,r}^{m}\right\}$$

where $x_{i,r}^{m}$ denotes the voxel sequence of the *r*-th ROI for the *i*-th subject under modality *m*. The length of this sequence is determined by the number of voxels in the current ROI on the target image grid:

(7) $$n_{i,r}^{m}=\left|\Omega_{i,r}^{m}\right|$$

where $n_{i,r}^{m}$ denotes the number of voxels in the *r*-th ROI under modality *m*. The voxels in the sequence are arranged according to a fixed traversal order on the target image grid. This process is applied to all sMRI feature maps and R-fMRI-derived local functional metric maps. Therefore, each imaging feature is converted from a whole-brain voxel map into 116 ROI voxel sequences.

For VMHC, its preprocessing includes symmetric template normalization. Therefore, its spatial information differs from that of ALFF, DC, and ReHo, which leads to inconsistent ROI voxel sequence lengths. To ensure batch processing and feature comparability, zero padding or truncation is used to unify the ROI voxel sequence length.

Through the above steps, each imaging feature is converted into ROI-level voxel sequences $x_{i,r}^{m}$. These sequences preserve intra-ROI voxel information and provide the direct inputs for ROI block construction and 3D ViT-based feature encoding in Section 3.3.2.

#### 3.3.2. ROI Voxel Organization and 3D ViT-Based Feature Encoding

Section 3.3.1 converts each imaging feature into ROI voxel sequences $x_{i,r}^{m}$. These sequences are one-dimensional representations. Directly using ROI mean values would discard fine-grained voxel information within each brain region, whereas directly feeding one-dimensional voxel sequences into the classifier would introduce high dimensionality and redundant information. Therefore, this subsection reorganizes each ROI voxel sequence into a three-dimensional ROI block and uses a 3D ViT-based ROI encoder to map it into a fixed-dimensional ROI representation. After this step, each ROI is represented by a feature vector $h_{i,r}^{m}$, which is used for multimodal fusion in Section 3.3.3.

Before constructing the three-dimensional ROI block, the ROI voxel sequence $x_{i,r}^{m}$ is first adjusted to a unified length. The length-aligned sequence is denoted as $x_{i,r}^{m}$, and its length is denoted as $l_{r}^{m}$. To construct a regular three-dimensional input, this study determines a near-cubic grid size $D_{r}^{m}\times H_{r}^{m}\times W_{r}^{m}$ for each ROI, satisfying:

(8) $$D_{r}^{m}\times H_{r}^{m}\times W_{r}^{m}\geq l_{r}^{m}$$

where $D_{r}^{m}$, $H_{r}^{m}$, and $W_{r}^{m}$ denote the depth, height, and width of the reconstructed ROI block, respectively. The sequence $x_{i,r}^{m}$ is then filled into this three-dimensional grid according to a fixed traversal order.

Because the voxel sequence is extracted according to a fixed traversal order on the preprocessed image grid, the same deterministic ordering is maintained during ROI block construction across subjects and imaging features. However, reshaping the one-dimensional voxel sequence into a near-cubic block does not strictly preserve the original three-dimensional anatomical neighborhood relationships within the ROI. Therefore, the reconstructed block should be regarded as a regularized ROI representation rather than an anatomically faithful reconstruction of the original ROI geometry. If the number of grid positions is larger than the sequence length, the remaining positions are filled with zeros. Z-score normalization is then performed within each ROI block. Through this step, the one-dimensional ROI voxel sequence is converted into a regularized three-dimensional ROI block:

(9) $$X_{i,r}^{m}\in R^{1\times D_{r}^{m}\times H_{r}^{m}\times W_{r}^{m}}$$

where the first dimension denotes the single-channel input. The resulting ROI block $X_{i,r}^{m}$ is used as the input of the 3D ViT-based ROI encoder.

Considering the variation in voxel numbers across ROIs, this study adopts a tiered adaptive strategy to set the patch size and encoder depth. Specifically, the 25th and 75th percentiles of ROI voxel numbers are used as thresholds to divide ROIs into small, medium, and large groups. Small ROIs use a patch size of 2×2×2, medium ROIs use a patch size of 4×4×4, and large ROIs use a patch size of 6×6×6. The corresponding encoder depths are set to 4, 6, and 6, respectively. This strategy balances local voxel granularity and token sequence length.

For a given ROI block, let the current patch size be $P_{r}^{m}\times P_{r}^{m}\times P_{r}^{m}$. The number of non-overlapping patches is defined as:

(10) $$K_{r}^{m}=\frac{D_{r}^{m}}{P_{r}^{m}}\frac{H_{r}^{m}}{P_{r}^{m}}\frac{W_{r}^{m}}{P_{r}^{m}},$$

where $K_{r}^{m}$ denotes the number of patches obtained from the *r*-th ROI under modality *m*. The *k*-th patch is denoted as $g_{i,r,k}^{m}$. This patch is first flattened and then projected into a *d*-dimensional token space:

(11) $$e_{i,r,k}^{m}=W_{E}\;\mathrm{vec}\left(g_{i,r,k}^{m}\right)+b_{E},$$

where $W_{E}$ and $b_{E}$ denote the projection matrix and bias term of patch embedding, respectively, and vec(·) denotes vectorization. In this study, *d* is set to 768. When eight attention heads are used, the dimension of each attention head is 96, which helps maintain regular head-wise dimension partitioning.

All patch embeddings are organized as a token sequence:

(12) $$E_{i,r}^{m}=\left[e_{i,r,1}^{m},e_{i,r,2}^{m},\ldots ,e_{i,r,K_{r}^{m}}^{m}\right].$$

Positional encoding ${\mathrm{PE}}_{r}^{m}$ is added to the patch token sequence to obtain the initial Transformer input $Z_{i,r,0}^{m}=E_{i,r}^{m}+{\mathrm{PE}}_{r}^{m}$, which is then input into the 3D ViT-based ROI encoder.

(13) $$Z_{i,r,l^{\prime }}^{m}=Z_{i,r,l-1}^{m}+\mathrm{MHSA}\left(\mathrm{LN}\left(Z_{i,r,l-1}^{m}\right)\right),$$

(14) $$Z_{i,r,l}^{m}=Z_{i,r,l^{\prime }}^{m}+\mathrm{FFN}\left(\mathrm{LN}\left(Z_{i,r,l^{\prime }}^{m}\right)\right),$$

where LN(·), MHSA(·), and FFN(·) denote layer normalization, multi-head self-attention, and the feed-forward network, respectively.

The ROI-level 3D ViT encoder does not use any external pretrained weights and is randomly initialized from scratch. Therefore, no additional pretraining dataset, pretraining objective, or pretrained checkpoint is involved in this study. The learnable positional embeddings are initialized using a truncated normal distribution with a standard deviation of 0.02, while the remaining modules use the default initialization schemes provided by PyTorch. A fixed global random seed is used to improve the reproducibility of the initialization and experimental procedure.

This study only uses the encoder part of the 3D ViT architecture as an ROI-level feature extractor. During supervised training, the encoder parameters are not updated. Accordingly, the ROI-level 3D ViT functions as a fixed nonlinear feature mapper that transforms ROI voxel blocks into fixed-dimensional ROI representations, rather than as an independently pretrained or MDD-specific representation learner. Keeping the encoder fixed also reduces the number of trainable parameters and avoids repeatedly optimizing the relatively large encoder during downstream feature-combination experiments.

After Transformer encoding, mean pooling is applied over all patch tokens to obtain the fixed-dimensional representation of the ROI:

(15) $$h_{i,r}^{m}=\frac{1}{K_{r}^{m}}\sum_{k=1}^{K_{r}^{m}}Z_{i,r,L,k}^{m},$$

where $Z_{i,r,L,k}^{m}$ denotes the final representation of the *k*-th patch token in the last Transformer layer for the *r*-th ROI of the *i*-th subject under imaging feature *m*, and $h_{i,r}^{m}\in R^{d}$ denotes the corresponding fixed-dimensional ROI representation. Through this process, each ROI voxel sequence is converted into a 768-dimensional ROI feature vector. For each imaging feature, all ROI vectors are further organized into an ROI-level feature matrix, which provides the direct input for multimodal ROI fusion in Section 3.3.3. Since the ROI-level 3D ViT encoder remains fixed during supervised training, these ROI representations can be precomputed during feature construction and reused across different feature-combination experiments. Therefore, this feature construction strategy reduces repeated ROI encoding costs and improves the overall efficiency of subsequent feature-combination analysis.

#### 3.3.3. Multimodal ROI Fusion and MLP Classification

Section 3.3.2 converts each imaging feature into fixed-dimensional ROI representations. This subsection further organizes these imaging-derived ROI representations and FC fingerprints within the same ROI semantic space, and uses an MLP classifier for MDD/NCs binary classification. The inputs of this subsection include the ROI-level feature matrices generated from sMRI feature maps and R-fMRI-derived local functional metric maps, as well as the ROI × ROI FC matrix obtained in Section 3.2. After ROI-level feature integration and MLP classification, the model outputs the predicted class probability of each subject belonging to NCs or MDD.

For the *i*-th subject, the representation of the *r*-th ROI under imaging feature *m* is denoted as $h_{i,r}^{m}$. By stacking all ROI representations according to the AAL atlas order, the ROI-level feature matrix of imaging feature *m* is obtained as:

(16) $$H_{i}^{m}={\left[h_{i,1}^{m},h_{i,2}^{m},\ldots ,h_{i,R}^{m}\right]}^{⊤}\in R^{R\times d},$$

where $H_{i}^{m}$ denotes the ROI-level feature matrix of the *i*-th subject under imaging feature *m*. In this way, each imaging feature is organized in the form of ROI × feature dimension.

For FC, Section 3.2 constructs an ROI × ROI FC matrix $C_{i}$ for each subject. The *r*-th row of this matrix represents the functional connectivity pattern between the *r*-th ROI and all other ROIs in the whole brain. Therefore, each row of the FC matrix is treated as the connectivity fingerprint of the corresponding ROI. Through this organization, FC is also converted into an ROI-level feature representation and remains consistent with imaging-derived ROI representations along the ROI dimension.

Let M denote the selected feature combination. For imaging features, $H_{i}^{m}$ is used as the ROI-level representation. For FC, $C_{i}$ is used as the ROI-level connectivity feature matrix. Under the same ROI order, the selected features are concatenated along the feature dimension to obtain the multimodal ROI-level fused feature of the *i*-th subject:

(17) $$F_{i}={\mathrm{Concat}}_{m\in M}\left(G_{i}^{m}\right)\in R^{R\times D_{M}},$$

where $G_{i}^{m}$ denotes the ROI-level feature matrix to be concatenated. When *m* is an imaging feature, $G_{i}^{m}=H_{i}^{m}$. When *m* is FC, $G_{i}^{m}=C_{i}$. $D_{M}$ denotes the total feature dimension after concatenating the selected features. The dimension of each imaging feature is *d*, whereas the dimension of the FC feature is *R*. This integration strategy first preserves brain-region correspondence along the ROI dimension and then concatenates the aligned feature representations along the feature dimension. In this way, structural features, local functional features, and FC fingerprints are jointly organized at the same brain-region semantic level before classification.

After multimodal ROI fusion, the fused feature matrix $F_{i}$ is flattened into a sample-level feature vector. The flattened feature vector is then standardized using Z-score normalization and input into the MLP classifier for binary classification. The MLP contains four hidden layers with widths of 640, 512, 384, and 256. Each hidden layer consists of a linear transformation, batch normalization, rectified linear unit (ReLU) activation, and dropout. The output layer contains two neurons corresponding to NCs and MDD.

Under the WM + FC + ReHo + DC configuration, the integrated ROI-level feature matrix has a size of 116×2420 and is flattened into a 280,720-dimensional input vector. The downstream MLP contains 180,289,282 trainable parameters. Counting multiplication and addition separately, the linear layers require approximately 0.361 GFLOPs per subject for a forward pass.

During training, the cross-entropy loss function is used:

(18) $$L=-\frac{1}{N}\sum_{i=1}^{N}\sum_{c=1}^{2}y_{i,c}\log\left({\hat{y}}_{i,c}\right),$$

where *N* denotes the number of training samples, $y_{i,c}$ denotes the one-hot ground-truth label of the *i*-th subject for class *c*, and ${\hat{y}}_{i,c}$ denotes the predicted probability that the *i*-th subject belongs to class *c*.

The MLP classifier is trained using the AdamW optimizer. The initial learning rate is set to $9\times 10^{-4}$, and the weight decay is set to $1\times 10^{-4}$. The learning-rate schedule uses 15 epochs of linear warmup followed by cosine annealing. The maximum number of training epochs is set to 400, and early stopping with a patience of 100 is used for model selection.

### 3.4. Experimental Settings and Evaluation Metrics

To evaluate the performance of MUSA on the MDD/NCs classification task, this study adopted a stratified hold-out strategy combined with five-fold cross-validation. All samples were first randomly divided into a training-validation set and an held-out test set at a ratio of 8:2, with the random seed fixed at 42. The full dataset contained 2380 subjects, including 1276 patients with MDD and 1104 NCs. The training-validation set contained 1904 subjects, including 1021 patients with MDD and 883 NCs. The held-out test set contained 476 subjects, including 255 patients with MDD and 221 NCs.

Five-fold cross-validation was then performed within the training-validation set. In each fold, four folds were used for training and one fold was used for validation. For each fold, the checkpoint with the best validation performance was retained, resulting in five fold-specific models. All five models were subsequently evaluated on the same held-out test set of 476 subjects. Therefore, the reported mean and standard deviation represent the variability in test performance across the five fold-specific models under the same fixed test conditions, rather than five independent test repetitions. During each fold, Z-score standardization was fitted only on the training subset and then applied to the corresponding validation and held-out test data to avoid information leakage.

Because the same held-out subjects were evaluated by the compared models, additional subject-level paired statistical analyses were performed. For the single-feature comparisons between MUSA and the baseline models, paired differences in ACC were reported together with 95% confidence intervals estimated using paired bootstrap resampling at the subject level, and McNemar’s test was used to assess paired differences in classification correctness. For the supplementary controlled comparisons involving ACC, AUC, and F1-score, 95% confidence intervals were estimated using class-stratified bootstrap resampling at the subject level. The same resampled subject indices were applied to the paired models while preserving each subject’s complete set of fold-specific predictions, and statistical significance was assessed using two-sided subject-level paired permutation tests. Raw *p*-values from the predefined comparisons were adjusted using the Holm procedure for multiple testing.

Model performance was evaluated using ACC, sensitivity (SEN), specificity (SPE), precision (PRE), F1-score (F1), and area under the receiver operating characteristic (ROC) curve (AUC). In this study, MDD was regarded as the positive class, and NCs were regarded as the negative class. Let TP, TN, FP, and FN denote true positive, true negative, false positive, and false negative, respectively. The evaluation metrics are defined as follows:

(19) $$\mathrm{ACC}=\frac{\mathrm{TP}+\mathrm{TN}}{\mathrm{TP}+\mathrm{TN}+\mathrm{FP}+\mathrm{FN}},$$

(20) $$\mathrm{SEN}=\frac{\mathrm{TP}}{\mathrm{TP}+\mathrm{FN}},$$

(21) $$\mathrm{SPE}=\frac{\mathrm{TN}}{\mathrm{TN}+\mathrm{FP}},$$

(22) $$\mathrm{PRE}=\frac{\mathrm{TP}}{\mathrm{TP}+\mathrm{FP}},$$

(23) $$F1=\frac{2\times \mathrm{PRE}\times \mathrm{SEN}}{\mathrm{PRE}+\mathrm{SEN}}.$$

AUC was used to evaluate the threshold-independent discriminative ability of the model and is defined as the area under the ROC curve:

(24) $$\mathrm{AUC}=\int_{0}^{1}\mathrm{TPR}\left(\mathrm{FPR}\right)\;d\mathrm{FPR},$$

where TPR denotes the true positive rate, and FPR denotes the false positive rate.

## 4. Results

### 4.1. Analysis and Comparison of Single-Feature Results

To evaluate the discriminative ability of different single features in the MDD/NCs binary classification task, this study conducted single-feature experiments on sMRI features, R-fMRI-derived local functional metrics, and FC. Three representative modeling methods were compared: ROI-Mean, 3D DenseNet121 [31], and MUSA. ROI-Mean represents ROI-level statistical modeling by averaging voxel values within each ROI. 3D DenseNet121 represents a whole-brain voxel-wise deep learning model. MUSA represents the proposed ROI-level voxel encoding model within a unified ROI semantic space.

As shown in Table 3, sMRI features achieved better performance than R-fMRI-derived local functional metrics and FC under MUSA. Among all single features, WM achieved the highest ACC, reaching 72.52%±1.27%. GM and CSF achieved ACC values of 72.35%±2.02% and 69.58%±0.82%, respectively. In contrast, the single-feature ACC values of ReHo, DC, ALFF, VMHC, and FC were lower than those of the structural features.

Compared with ROI-Mean, MUSA achieved higher ACC values on all sMRI features and R-fMRI-derived local functional metrics. Compared with 3D DenseNet121, MUSA also showed higher or comparable ACC values on most single-feature channels. Overall, the single-feature results indicate that structural features provide stronger individual discriminative ability under the current experimental setting. The numerical comparisons also suggest that the ROI-level voxel representation of MUSA provides competitive classification performance relative to ROI-Mean and whole-brain 3D DenseNet121 across different feature channels. R-fMRI-derived local functional metrics and FC showed limited discriminative ability when used alone, but they may provide complementary information for subsequent multimodal modeling.

Additional subject-level paired comparisons were performed between MUSA and the ROI-Mean and 3D DenseNet121 baselines for the seven feature types applicable to all three methods. MUSA showed positive ACC differences relative to both baselines across all evaluated feature types. After Holm correction, statistically significant differences remained between MUSA and ROI-Mean for GM (ΔACC = +14.71 percentage points, adjusted *p* < 0.001), ReHo (+5.04, adjusted *p* = 0.047), DC (+7.98, adjusted *p* = 0.004), and VMHC (+6.51, adjusted *p* = 0.016), and between MUSA and 3D DenseNet121 for WM (+7.56, adjusted *p* = 0.004) and VMHC (+6.72, adjusted *p* = 0.009). The remaining numerical differences did not remain statistically significant after multiple-comparison correction. The complete paired statistical results are provided in Supplementary Table S1.

### 4.2. Comparison of Feature Combinations Within the Same Modality

After the single-feature analysis, this study further evaluated feature combinations within the same modality. The aim was to examine whether features from the same modality could provide complementary information. The sMRI combinations were used to assess the joint effect of different tissue components, while the R-fMRI combinations were used to assess the joint effect of local functional metrics and FC.

#### 4.2.1. Results of Feature Combinations Within the sMRI Modality

As shown in Table 4, the sMRI feature combinations showed relatively stable performance. However, their numerical improvement over the strongest single structural feature was limited. The single feature with the highest ACC in Section 4.1 was WM, with an ACC of 72.52%±1.27%. In comparison, among the evaluated sMRI combinations, GM + WM + CSF achieved an ACC of 72.44%±0.93%.

Although GM + WM + CSF did not increase ACC over WM alone, it showed a smaller standard deviation. This numerical pattern suggests that combining structural channels may improve performance stability rather than overall accuracy. Among the two-feature combinations, GM + WM achieved the highest ACC of 72.31%±1.47%, while GM + CSF and WM + CSF achieved lower ACC values. Overall, joint modeling within the sMRI modality showed limited additional gain over the strongest single structural feature, although some combinations exhibited lower performance variability.

Overall, joint modeling within the sMRI modality can improve result stability to a certain extent. However, relying only on structural information still provides limited additional gain over the discriminative performance of a strong single structural channel.

#### 4.2.2. Results of Feature Combinations Within the R-fMRI Modality

Within the R-fMRI modality, this study further compared combinations of local functional metrics and the performance changes after introducing FC. As shown in Table 5, feature combinations within the R-fMRI modality generally achieved higher numerical performance than single functional metrics, although clear differences remained among different combinations.

Among the combinations without FC, DC + ALFF + VMHC achieved the highest ACC among the evaluated configurations, with an ACC of 65.59%±1.62%, an AUC of 71.03%±1.33%, and an F1 of 68.42%±2.04%. In contrast, after adding ReHo, the ACC of ReHo + DC + ALFF + VMHC decreased to 65.04%±1.14%, and the AUC decreased to 70.13%±1.26%. This suggests that the effect of combining local functional metrics does not simply increase with the number of features.

Among the combinations including FC, FC + ReHo + ALFF + VMHC achieved the highest ACC among the evaluated configurations, with an ACC of 66.51%±0.82%, an AUC of 73.19%±0.69%, and an F1 of 69.38%±0.46%. This configuration showed higher numerical performance and a smaller standard deviation than DC + ALFF + VMHC, which achieved the highest ACC among the evaluated combinations without FC. These numerical results suggest that FC may provide complementary information to local functional metrics within the current experimental setting.

### 4.3. Multimodal Fusion and Combination Ablation Results

After the single-feature and within-modality combination analyses, this study further evaluated the effect of cross-modality feature combinations on MDD/NCs classification performance. To systematically examine the effects of feature composition, all 255 non-empty combinations of the eight candidate feature types, including GM, WM, CSF, FC, ReHo, DC, ALFF, and VMHC, were evaluated under the same data split, model architecture, training strategy, and evaluation protocol. No additional hyperparameter tuning, feature weighting, or combination-specific optimization was performed for any individual feature configuration. For readability, Table 6 presents representative WM-based multimodal combinations from the systematically evaluated feature-combination space.

As shown in Table 6, when WM was used as the structural feature basis, the mean ACC generally increased as local functional metrics and FC were progressively introduced. WM + ReHo achieved an ACC of 77.14%±0.87% and an AUC of 86.66%±1.22%. After DC was added, the ACC of WM + ReHo + DC increased to 77.82%±1.23%. This numerical pattern suggests that different local functional metrics may provide additional discriminative information beyond structural information.

After FC was further introduced, WM + FC + ReHo achieved an ACC of 78.45%±0.57% and an AUC of 87.39%±0.69%. Compared with WM + ReHo, this combination showed a higher mean ACC and a smaller ACC standard deviation. These numerical results suggest that the interregional connectivity patterns represented by FC may provide complementary information to local functional metrics.

Among the systematically evaluated feature combinations, WM + FC + ReHo + DC achieved the highest mean ACC under the current evaluation protocol. This combination obtained an ACC of 79.20%±0.65%, SEN of 83.22%±2.35%, SPE of 74.57%±3.92%, AUC of 87.08%±0.51%, and F1 of 81.09%±0.29%. These numerical results indicate that joint modeling of structural features, local functional metrics, and FC can achieve favorable classification performance under the current experimental setting. However, the observed differences among the representative feature combinations should be interpreted descriptively rather than as evidence of statistically significant superiority. The performance variations across different combinations further suggest that integrating multi-level MRI information within a unified ROI semantic space may provide complementary discriminative information for MDD/NCs classification.

To further assess the key feature-combination comparisons, two additional feature-removal controls were evaluated by removing FC or DC from the WM + FC + ReHo + DC configuration. Compared with these newly trained controls, the historical main MUSA model showed mean ACC differences of 0.80 percentage points versus WM + ReHo + DC and 0.97 percentage points versus WM + FC + ReHo, with paired 95% confidence intervals of −0.50 to 2.14 and −0.34 to 2.35 percentage points, respectively. Across the predefined ACC, AUC, and F1 comparisons, none of the differences remained statistically significant after Holm correction. The complete paired statistical results and the sources of the supplementary control models are provided in the Supplementary Materials.

The above results indicate that MRI feature type and modality composition affect classification performance. When used individually, structural MRI features, particularly WM and GM, showed stronger discriminative performance than individual R-fMRI-derived local functional metrics and FC. When functional information was combined with structural features, several multimodal configurations showed higher numerical performance than the corresponding structural-only or lower-order combinations. In particular, the combinations incorporating ReHo, DC, and FC with WM achieved relatively high ACC values under the current evaluation protocol. Therefore, these results should not be interpreted as demonstrating the absolute superiority of one modality or feature combination over another. Rather, structural and functional MRI-derived features may provide different and complementary sources of discriminative information, and their joint organization within the unified ROI semantic space can support MDD/NCs classification under the current experimental setting.

Additional analysis of ROI spatial representation. To further examine the potential influence of the ROI reshaping strategy, we conducted an additional experiment using a coordinate-preserving ROI representation under the WM + FC + ReHo + DC configuration. In this representation, ROI voxels were placed according to their spatial coordinates in the preprocessed image grid within the minimum ROI bounding box, while non-ROI locations were zero-padded. FC representation remained unchanged. The coordinate-preserving representation achieved an ACC of 71.72%, an AUC of 77.54%, and an F1-score of 74.48%. The coordinate-preserving representation did not yield higher classification performance than the near-cubic representation under the current controlled setting. However, because the alternative representation also changes the input extent, zero-padding ratio, token number, and positional organization, the observed performance difference should not be attributed solely to the preservation of anatomical spatial relationships. Detailed experimental settings and statistical results are provided in the Supplementary Materials.

Additional analysis of adaptive patch-size and encoder-depth configuration. The adaptive configuration was further compared with three fixed patch-size/encoder-depth configurations.

As shown in Table 7, the adaptive configuration achieved the highest ACC and F1-score among the evaluated configurations, whereas the fixed-medium configuration achieved a slightly higher numerical AUC. Because patch size and encoder depth varied jointly in this comparison, the observed differences cannot be attributed solely to either factor.

Additional analysis of encoder depth. To further isolate the effect of encoder depth, the patch size was fixed at 4×4×4 and only the encoder depth was varied.

As shown in Table 8, ACC, F1-score, and AUC increased progressively as the encoder depth increased from 2 to 6. The improvement from Depth-2 to Depth-4 was larger than that from Depth-4 to Depth-6, indicating a smaller marginal gain at greater depth under the current setting.

Additional analysis of multimodal fusion strategy. The current feature-concatenation strategy was further compared with late fusion and attention-based fusion.

As shown in Table 9, feature concatenation achieved the highest ACC and F1-score among the three fusion strategies, whereas attention fusion achieved a slightly higher numerical AUC. Late fusion showed lower performance across all three metrics. These results indicate that the current feature-concatenation strategy provides competitive classification performance under the present experimental setting.

### 4.4. Comparison Experiments

To evaluate the effectiveness of MUSA in the MDD/NCs classification task, this study compared it with three categories of representative methods: ROI-level representation methods, whole-brain deep learning models, and randomly initialized front-end encoding methods. These methods were used to examine the effects of different ROI feature construction strategies, compare ROI-level organized modeling with whole-brain voxel-wise modeling, and determine whether the performance advantage of MUSA came only from the front-end encoder or from the joint effect of unified ROI semantic organization, ROI-level feature encoding, and multimodal fusion. All methods used the same data split and evaluation metrics described in Section 3.4 to ensure fair comparison.

The ROI-level baselines included ROI-Mean + MLP, ROI-PCA + MLP, and ROI-Vector + MLP. ROI-Mean + MLP used the mean value within each ROI as the brain-region feature. ROI-PCA + MLP applied principal component analysis to intra-ROI voxel features. ROI-Vector + MLP directly used vectorized ROI features. The randomly initialized baselines included Random 3D CNN + MLP, Random 3D ViT + Linear classifier, and Random 3D ViT + SVM. Multi-stream 3D DenseNet121 and 3D RepViT were used as whole-brain deep learning baselines. Because the complete source code of the original Multi-stream 3D DenseNet121 and 3D RepViT studies was not publicly available, this study reproduced them according to the network structures, input forms, and training strategies provided in the literature, and evaluated them under the unified experimental setting.

Table 10 presents the overall classification results of different methods. Under the WM + FC + ReHo + DC configuration, MUSA achieved an ACC of 79.20%±0.65%, SEN of 83.22%±2.35%, SPE of 74.57%±3.92%, AUC of 87.08%±0.51%, and F1 of 81.09%±0.29%. Among the compared methods, MUSA obtained the highest mean ACC, AUC, and F1.

Compared with ROI-level representation methods, MUSA achieved higher numerical ACC and AUC values. ROI-Mean + MLP and ROI-PCA + MLP achieved relatively low ACC and AUC values, whereas ROI-Vector + MLP increased the ACC to 73.11%±1.23% and the AUC to 83.93%±1.36%. These comparisons suggest that preserving richer intra-ROI voxel information may be beneficial relative to simple ROI averaging or linear dimensionality reduction. Compared with whole-brain deep learning models, MUSA also achieved higher numerical performance than Multi-stream 3D DenseNet121 and 3D RepViT under the current experimental setting. This result suggests that explicit ROI-level semantic organization may provide a useful alternative to direct whole-brain voxel-wise modeling for this task. In addition, Random 3D ViT + Linear classifier and Random 3D ViT + SVM achieved moderate ACC values, but their AUC values were lower than that of MUSA. These results suggest that the observed performance of MUSA cannot be attributed solely to the use of a 3D ViT front-end, and may also reflect the joint contribution of ROI-level feature encoding, unified ROI semantic organization, and multimodal feature integration.

To further examine the effect of updating the ROI-level 3D ViT encoder during supervised training, an additional Random-Trainable control experiment was conducted under the WM + FC + ReHo + DC configuration. Both settings used random initialization without external pretraining and differed only in whether the encoder parameters were updated during downstream supervised training.

As shown in Table 11, jointly optimizing the randomly initialized 3D ViT encoder did not improve classification performance under the current data scale and training setting. The observed performance decrease provides empirical support for retaining the frozen encoder in the present implementation, but should not be interpreted as evidence that freezing is universally preferable to encoder adaptation.

**Figure 4:**
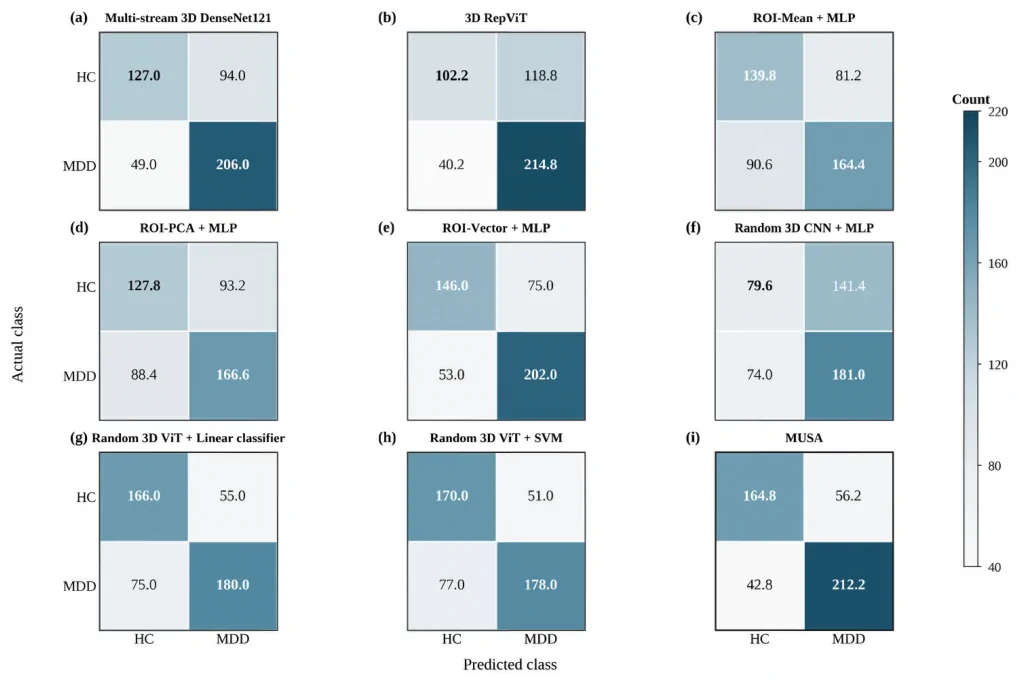
Average confusion matrices of different methods.

Figure 4 shows the average confusion matrices of the compared methods. MUSA correctly classified an average of 164.8 NCs and 212.2 MDD samples across the five fold-specific models. The corresponding error distribution is consistent with the relatively high SEN and F1 values reported in Table 10 and indicates that the model maintained effective recognition of MDD samples under the current evaluation setting.

**Figure 5:**
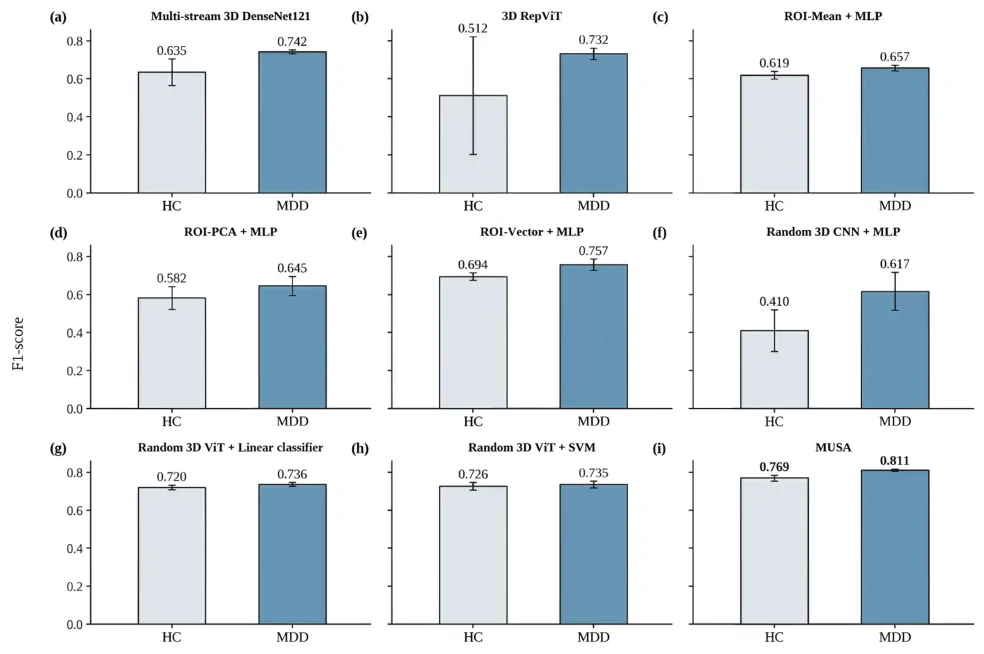
Class-level F1-scores of different methods.

Figure 5 presents the F1-scores of different methods for the NCs and MDD classes. MUSA achieved an F1-score of 76.85%±1.42% for NCs and 81.09%±0.29% for MDD, which were numerically higher than those of the comparison methods overall. These class-level results indicate that MUSA maintained relatively balanced classification performance across NCs and MDD.

**Figure 6:**
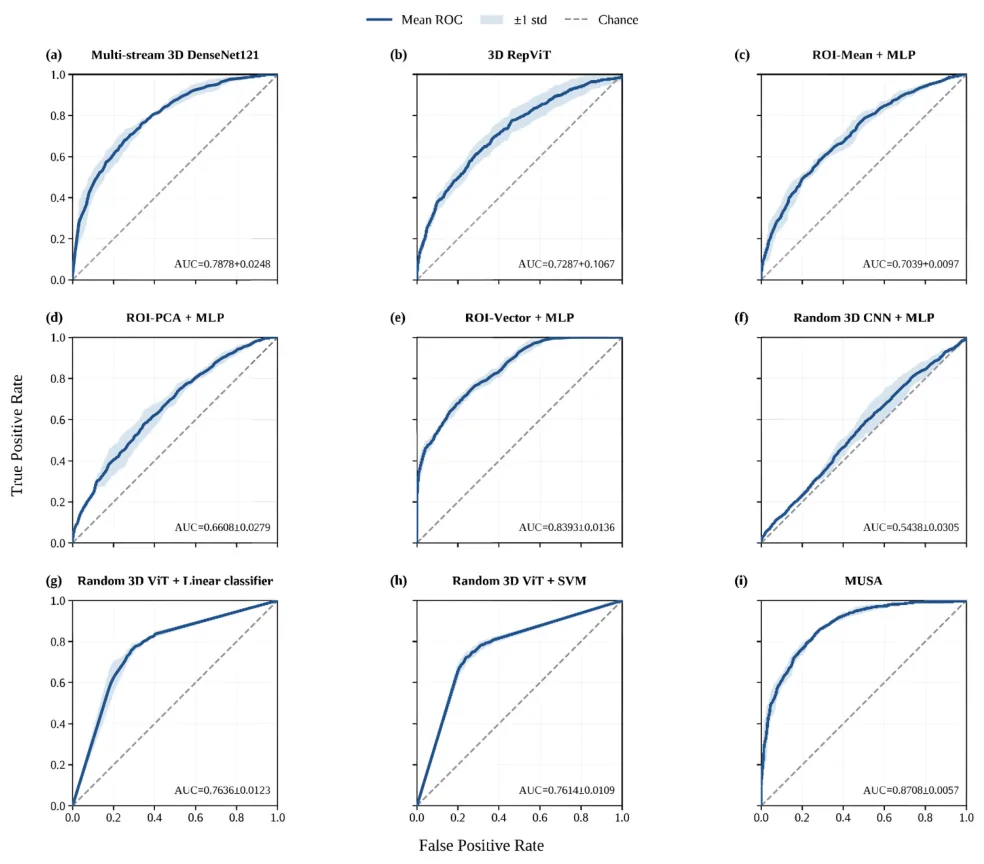
ROC curves of different methods.

Figure 6 shows the ROC curves of different methods. The mean ROC curve of MUSA was generally above those of the other methods, and its AUC reached 87.08%±0.51%, which was the highest among all comparison methods. In contrast, the ROI-level statistical methods and the random convolutional front-end method showed weaker ROC performance. The Random 3D ViT-based methods also achieved lower AUC values than MUSA. These numerical differences indicate stronger threshold-independent discriminative performance for MUSA under the current evaluation setting.

Failure-case analysis. To further examine subject-level prediction stability, the predictions of the five fold-specific models under the WM + FC + ReHo + DC configuration were analyzed on the same held-out test set of 476 subjects. Among these subjects, 303 (63.66%) were correctly classified by all five models, 34 (7.14%) were misclassified by all five models, and the remaining 139 (29.20%) showed prediction disagreement across the fold-specific models. The consistently misclassified subjects included 13 patients with MDD and 21 NCs. These results show that a substantial proportion of subjects were classified consistently across the five fold-specific models, while a smaller subset remained persistently difficult to classify or sensitive to variations in the training folds.

Overall, Table 10 and Figure 4, Figure 5 and Figure 6 show that MUSA achieved favorable numerical performance across overall accuracy, class-level F1-score, error distribution, and ROC-based discrimination. Compared with the evaluated ROI-level representation methods, whole-brain deep learning models, and randomly initialized front-end encoding methods, MUSA obtained higher values on several major evaluation metrics under the current experimental protocol. These findings support the usefulness of organizing ROI-level representations and multimodal MRI information within a unified ROI semantic space for MDD/NCs classification.

### 4.5. Analysis of Input Dimensionality and Classifier Capacity

To further examine the influence of the high-dimensional flattened representation and classifier capacity, we conducted additional controlled experiments under the WM + FC + ReHo + DC configuration. In addition to the original MUSA classifier, we evaluated a parameter-matched control model, a 1024-dimensional compressed-input baseline, and a reduced-capacity MLP while retaining the full fused input. As described in Section 3.3.3, the original fused ROI-level feature matrix has a size of 116×2420 and is flattened into a 280,720-dimensional input vector. The downstream MLP contains 180,289,282 trainable parameters, and its linear layers require approximately 0.361 GFLOPs per subject for a forward pass when multiplication and addition are counted separately. The results of these controlled experiments are summarized in Table 12.

The original MUSA model achieved an ACC of 79.20%, an AUC of 87.08%, and an F1-score of 81.09%. The parameter-matched control model achieved 75.34%, 83.19%, and 77.03%, respectively. The 1024-dimensional compressed-input baseline achieved an ACC of 71.60%, an AUC of 79.31%, and an F1-score of 73.42%, whereas the reduced-capacity MLP achieved 78.74%, 88.14%, and 80.77%, respectively. Although the reduced-capacity MLP achieved a slightly higher numerical AUC than the original MUSA classifier, its ACC and F1-score remained close to those of the original model.

Subject-level paired analyses were performed using stratified bootstrap confidence intervals and paired permutation tests with Holm correction. After multiple-comparison correction, significant differences were observed between MUSA and both the parameter-matched control model and the 1024-dimensional compressed-input baseline, whereas the differences between MUSA and the reduced-capacity MLP did not remain statistically significant.

These results suggest that aggressive dimensionality reduction may discard useful discriminative information contained in the fused ROI representation. The lower performance of the parameter-matched control indicates that the performance of MUSA cannot be explained solely by classifier parameter count. At the same time, the reduced-capacity MLP retained performance close to that of the original classifier, with no statistically significant difference after multiple-comparison correction. Therefore, the current four-layer MLP should not be interpreted as a minimal or indispensable classifier design. These findings indicate that the unified ROI representation contains useful discriminative information while leaving room for further reduction of classifier complexity through more compact projection layers, learnable dimensionality-reduction strategies, or lightweight classifiers.

### 4.6. Analysis of Important Brain-Region Pairs in the Unified ROI Semantic Space

Based on the preceding AAL atlas-guided ROI parcellation and unified ROI semantic organization, MUSA organizes multimodal MRI information at explicit anatomical brain-region levels. Unlike models that directly take whole-brain images as input, this design allows the discriminative information learned by the model to be traced back to specific ROIs and their left–right homologous brain-region pairs, thereby providing brain-region-level interpretability for the classification results.

Based on this advantage, this study was able to further analyze the importance of each ROI in the unified ROI semantic space and construct a ranking of important brain-region pairs. The results showed that the discriminative information used by MUSA for MDD/NCs classification was not concentrated in a single brain region, but distributed across frontoparietal control, sensorimotor, visual, temporal association, auditory/language, and limbic-related functional domains. Importantly, these importance weights quantify the relative contribution of ROI-level features to the classifier decision and should not be interpreted as causal biomarkers, disease-specific abnormalities, or pathological mechanisms of MDD. The relatively high importance assigned to Frontal_Sup, Frontal_Inf_Oper, Frontal_Mid_Orb, and Parietal_Inf were consistent with previous MDD findings related to abnormalities in prefrontal, orbitofrontal, and executive-control-related regions [40,41]. In addition, the presence of Postcentral, Precentral, Occipital_Inf, Temporal_Inf, Temporal_Sup, and ParaHippocampal suggests that the discriminative patterns captured by MUSA further extended to sensorimotor, visual, temporal, and limbic memory-related systems. This finding is consistent with large-scale and cross-modal studies suggesting that MDD involves abnormalities across multiple brain regions and functional systems [23,42,43].

Specifically, this study calculated the importance of each ROI based on the weights of the trained classifier. Because multimodal features had already been organized into the unified ROI semantic space, these weights could be mapped back to the corresponding brain-region units. Considering the natural anatomical correspondence between left and right homologous brain regions, and the fact that unilateral rankings may be affected by local fluctuations, this study further aggregated left and right homologous ROIs and used bilateral brain-region pairs as the analysis unit. This strategy reduced the influence of unilateral weight fluctuations and allowed the discriminative pattern of the model to be presented in a more stable brain-region-pair form.

**Figure 7:**
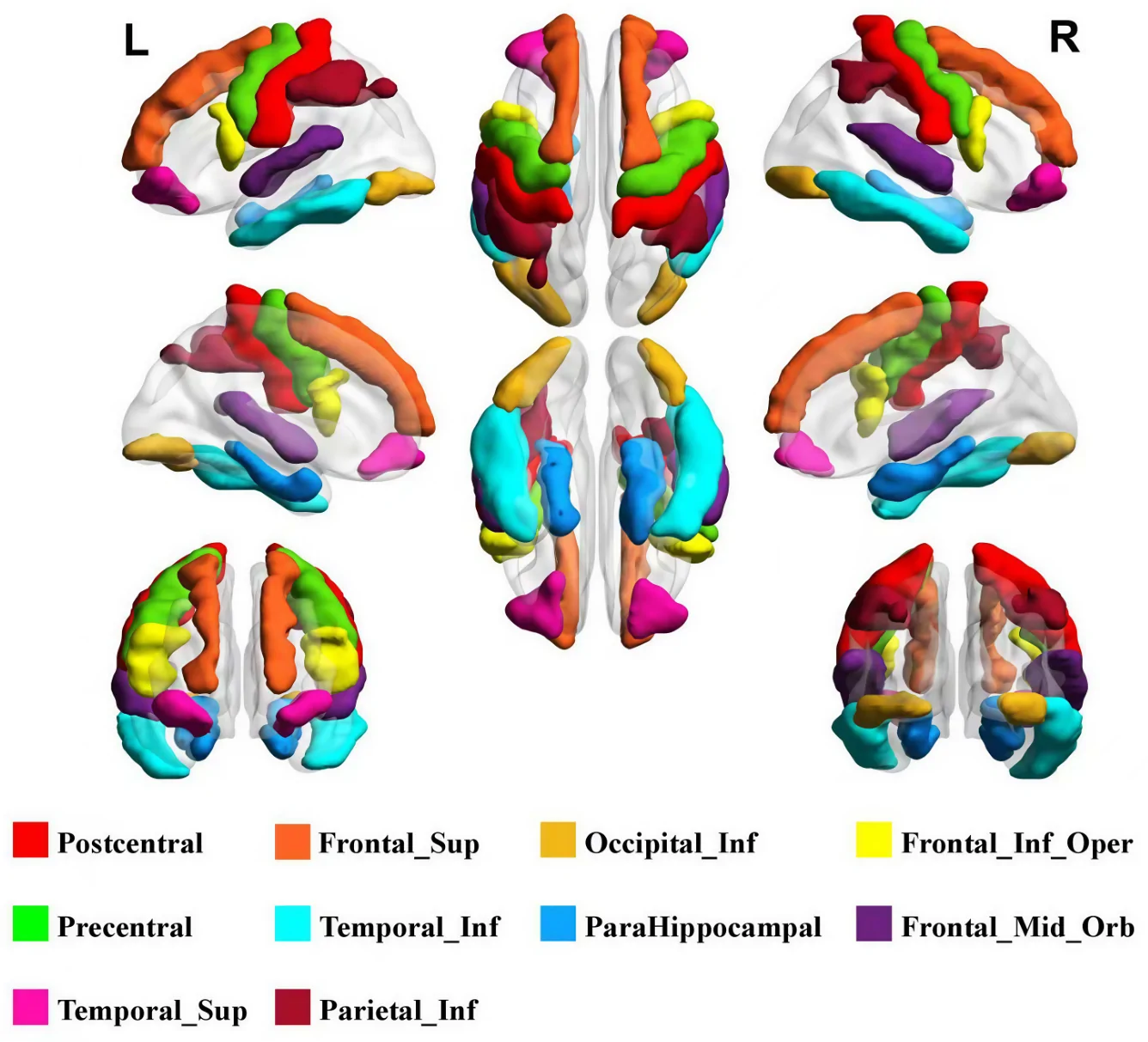
Spatial distribution of the top 10 important brain-region pairs.

Figure 7 shows the spatial distribution of the top 10 important brain-region pairs on the brain surface. Table 13 further summarizes their functional domains, mean importance weights, coefficients of variation, and left–right differences.

As shown in Figure 7 and Table 13, the top-ranked brain-region pairs included Postcentral, Frontal_Sup, Occipital_Inf, Frontal_Inf_Oper, and Precentral. These regions were mainly distributed in sensorimotor and frontoparietal control systems, with additional involvement of visual, temporal, auditory/language, and limbic-related regions. This distribution suggests that MUSA may use joint discriminative information across multiple functional systems rather than relying on a single local brain region.

From the perspective of previous studies, the high importance of Frontal_Sup, Frontal_Inf_Oper, Frontal_Mid_Orb, and Parietal_Inf is consistent with reported abnormalities in prefrontal, orbitofrontal, and executive-control-related regions in MDD [40,41]. Meanwhile, the presence of Postcentral, Precentral, Occipital_Inf, Temporal_Inf, Temporal_Sup, and ParaHippocampal indicates that the discriminative pattern captured by MUSA extended beyond the traditional prefrontal-limbic system. This finding is consistent with large-scale and cross-modal evidence suggesting that MDD may involve coordinated abnormalities across multiple brain regions and functional systems [23,44]. Nevertheless, such overlap does not establish that these regions constitute pathological biomarkers of MDD; rather, it provides external neuroimaging context for interpreting the classifier-derived spatial pattern.

Overall, these classifier-derived importance patterns should not be interpreted as causal evidence or pathological mechanisms of MDD. Instead, they provide brain-region-level interpretive evidence regarding which ROI-level features contributed more strongly to the classification decisions of MUSA. The preceding ROI parcellation and unified ROI semantic organization also allow these discriminative patterns to be mapped back to specific ROIs and homologous brain-region pairs, providing an anatomically interpretable representation of the model’s classification decisions.

### 4.7. Discussion and Limitations

Although REST-meta-MDD provides a relatively large multicenter neuroimaging dataset, the current study used a subject-level stratified split rather than a site-held-out evaluation. Therefore, subjects from the same acquisition site may appear in both the training and held-out test sets, and the present results should primarily be interpreted as internal validation within REST-meta-MDD rather than evidence of generalization to unseen centers or scanners. Standardized preprocessing and AAL-based ROI alignment may reduce part of the variability across subjects, but they do not explicitly remove site-related distribution shifts. Future studies should therefore include independent external cohorts and leave-one-site-out validation.

The held-out test set was separated from the training-validation samples and was not used for model parameter optimization. However, because multiple feature combinations were evaluated on the same held-out set, it also served as a common comparison set for feature selection. Thus, it should not be regarded as a strictly untouched final test set in this respect. In addition, the reported mean and standard deviation reflect variability across five fold-specific models evaluated on the same test subjects rather than independent test repetitions.

The current framework also depends on several design choices. The ROI-level 3D ViT is randomly initialized and frozen during downstream training, and should therefore be interpreted as a fixed nonlinear feature mapper rather than an independently learned MDD-specific encoder. The near-cubic ROI reconstruction does not strictly preserve the original anatomical neighborhood structure, while the use of the AAL atlas makes ROI representations and FC construction atlas-dependent. Different parcellation schemes, such as the Schaefer functional atlas, may alter ROI boundaries, voxel composition, representation quality, and the resulting FC patterns, thereby affecting downstream classification performance. The robustness of MUSA across anatomical and functional parcellation schemes therefore remains to be systematically evaluated. In addition, the high-dimensional fused representation leads to a relatively large downstream MLP. Supplementary analyses showed that strong dimensionality reduction reduced performance, whereas a reduced-capacity MLP retained comparable performance, suggesting that the current classifier may still be further simplified.

A strict single-factor ablation of the ROI semantic organization itself was not performed because removing or replacing this organization would simultaneously alter the cross-modal regional correspondence, input arrangement, and subsequent fusion structure. Therefore, its independent contribution remains to be disentangled more rigorously in future work. Similarly, the current experiments evaluate the contribution of FC information but do not independently compare alternative FC fingerprint representations; therefore, the superiority of the present FC representation scheme over other possible formulations has not been established.

The ROI importance analysis should be interpreted strictly from a model-explainability perspective. The reported importance weights represent the relative contribution of ROI-level features to classifier decisions and should not be regarded as causal biomarkers or direct evidence of MDD pathological mechanisms. Similarly, the failure-case analysis showed that aggregate metrics do not fully characterize subject-level prediction stability: some subjects were consistently classified correctly, whereas others were persistently misclassified or showed disagreement across folds.

Another limitation is that predictive uncertainty was not explicitly quantified. Although the classifier outputs class probabilities, these probabilities should not necessarily be interpreted as calibrated confidence estimates, as uncertainty estimation and probability calibration remain important considerations for reliable deep learning applications [45,46].

The relatively straightforward MLP was adopted to minimize additional structural assumptions and to more directly evaluate the discriminative utility of the unified ROI semantic representation. The current downstream classifier does not explicitly model graph-structured interregional dependencies. Since each ROI can naturally be treated as a graph node and the available FC fingerprints can characterize interregional relationships, future work may explore ROI-graph-based classifiers, such as graph convolutional networks, graph attention networks, or Graph Transformers.

## 5. Conclusions

This study proposed MUSA, a multimodal MRI-based major depressive disorder classification model with a unified ROI semantic space, for auxiliary classification between MDD and NCs. Guided by the AAL atlas, MUSA organizes sMRI features, R-fMRI-derived local functional metrics, and FC information at a consistent brain-region semantic level, while preserving intra-ROI voxel information and integrating interregional connectivity patterns. Experimental results on the large-scale REST-meta-MDD dataset showed that MUSA achieved competitive MDD/NCs classification performance under the current evaluation protocol. The WM + FC + ReHo + DC configuration achieved the highest mean ACC among the evaluated feature combinations, with an ACC of 79.20%±0.65% and an AUC of 87.08%±0.51%.

Further brain-region importance analysis showed that the classifier-derived discriminative information was distributed across frontoparietal, sensorimotor, visual, temporal, and limbic-related functional domains. These importance patterns reflect the contribution of ROI-level features to the classifier decision and should not be interpreted as causal biomarkers or pathological mechanisms of MDD. The results indicate that the unified ROI semantic space can support multimodal MRI feature organization while retaining brain-region-level interpretability.

Future work will further evaluate the classification performance and generalization ability of MUSA using independent external cohorts and site-independent validation, and will investigate alternative brain parcellations, more compact classifiers, graph-based ROI modeling, uncertainty calibration, and the integration of clinical and demographic information.

## Figures and Tables

**Table 2:** Demographic information of the included participants.

				Average ± Standard Deviation (Years)	
Group	Number of Participants	Male	Female	Age	Years of Education
MDD	1276	463	813	36.23±14.62	11.25±4.21
NCs	1104	462	642	36.15±15.66	12.25±4.98

**Table 3:** Accuracy of single features under the three methods.

		Average ± Standard Deviation (%)		
Modality	Single-Feature Channel	MUSA	ROI-Mean	3D DenseNet121
sMRI	GM	72.35±2.02	57.73±1.31	68.24±3.80
	WM	72.52±1.27	68.61±1.92	64.92±6.18
	CSF	69.58±0.82	67.31±1.02	65.55±3.38
R-fMRI	ReHo	60.55±1.57	55.42±1.87	59.41±1.88
	DC	60.63±2.47	52.73±1.62	56.01±1.23
	ALFF	60.00±1.89	55.42±2.28	55.17±2.66
	VMHC	62.06±2.78	55.55±1.03	55.38±2.78
	FC	60.25±1.07	—	—

Note: “—” indicates that the method is not applicable to this input form.

**Table 4:** Classification results of different sMRI feature combinations.

	Average ± Standard Deviation (%)				
Feature	ACC	SEN	SPE	AUC	F1
GM + WM	72.31±1.47	76.94±3.53	66.97±4.20	79.87±1.25	74.84±1.53
GM + CSF	69.71±1.80	71.53±4.08	67.60±2.85	78.78±1.34	71.63±2.28
WM + CSF	71.89±1.75	80.00±3.63	62.53±3.47	80.52±2.00	75.28±1.79
GM + WM + CSF	72.44±0.93	76.55±1.76	67.69±3.06	80.72±1.16	74.85±0.71

**Table 5:** Classification results of different channel combinations within the R-fMRI modality.

	Average ± Standard Deviation (%)				
Feature	ACC	SEN	SPE	AUC	F1
ALFF + VMHC	63.24±1.84	68.08±7.06	57.65±6.65	69.08±1.65	66.37±2.92
DC + ALFF + VMHC	65.59±1.62	69.73±4.68	60.81±5.57	71.03±1.33	68.42±2.04
ReHo + DC + ALFF + VMHC	65.04±1.14	67.29±6.44	62.44±9.33	70.13±1.26	67.26±1.64
FC + ALFF	63.82±1.52	69.10±4.12	57.74±4.10	70.60±1.86	67.13±2.03
FC + ReHo + ALFF + VMHC	66.51±0.82	70.82±1.16	61.54±2.70	73.19±0.69	69.38±0.46
FC + ReHo + DC + ALFF + VMHC	65.08±1.80	69.65±5.03	59.82±8.88	71.83±0.83	68.09±1.18

**Table 6:** Representative results of multimodal combination ablation.

	Average ± Standard Deviation (%)				
Feature	ACC	SEN	SPE	AUC	F1
WM + ReHo	77.14±0.87	81.02±4.53	72.67±3.95	86.66±1.22	79.12±1.48
WM + ReHo + DC	77.82±1.23	80.47±1.99	74.75±4.10	86.67±1.96	79.54±0.85
WM + FC + ReHo	78.45±0.57	81.88±3.52	74.48±4.26	87.39±0.69	80.26±0.78
WM + FC + ReHo + DC	79.20±0.65	83.22±2.35	74.57±3.92	87.08±0.51	81.09±0.29

**Table 7:** Ablation of patch-size and encoder-depth configurations.

Configuration	Patch Size	Encoder Depth	ACC (%)	F1-Score (%)	AUC (%)
MUSA (adaptive configuration)	Adaptive (2/4/6)	Adaptive (4/6/6)	79.20±0.65	81.09±0.29	87.08±0.51
Fixed-small configuration	2×2×2	4	71.58	72.97	83.01
Fixed-medium configuration	4×4×4	6	77.00	78.12	87.38
Fixed-large configuration	6×6×6	6	75.92	77.08	86.87

**Table 8:** Ablation of encoder depth.

Variant	Patch Size	Encoder Depth	ACC (%)	F1-Score (%)	AUC (%)	STD(ACC) (%)
Depth-2	4×4×4	2	74.16	75.32	85.07	4.15
Depth-4	4×4×4	4	76.05	77.26	86.59	3.42
Depth-6	4×4×4	6	77.00	78.12	87.38	3.86

**Table 9:** Ablation of multimodal fusion strategy.

Fusion Strategy	ACC (%)	F1-Score (%)	AUC (%)	STD (ACC) (%)
Feature concatenation	79.20	81.09	87.08	0.65
Late fusion	66.23	61.98	77.19	1.55
Attention fusion	78.20	80.30	87.10	1.55

**Table 10:** Comparison results of different models.

		Average ± Standard Deviation (%)				
Feature	Method	ACC	SEN	SPE	AUC	F1
WM + DC + ReHo	Multi-stream 3D DenseNet121	69.96±1.92	80.78±5.45	57.47±9.93	78.87±2.22	74.22±0.78
GM + WM	3D RepViT	66.60±6.86	84.24±8.36	46.24±23.31	72.87±9.54	73.18±2.56
WM + FC + ReHo + DC	ROI-Mean + MLP	63.91±1.27	64.47±2.94	63.26±3.42	70.39±0.97	65.66±1.56
	ROI-PCA + MLP	61.85±2.53	65.33±8.38	57.83±9.84	66.08±2.79	64.53±3.87
	ROI-Vector + MLP	73.11±1.23	79.22±9.93	66.06±7.91	83.93±1.36	75.72±3.32
	Random 3D CNN + MLP	54.75±3.20	70.98±18.37	36.02±14.07	54.38±3.05	61.75±8.65
	Random 3D ViT + Linear Classifier	72.82±0.62	70.67±2.90	75.29±3.69	76.36±1.23	73.57±0.72
	Random 3D ViT + SVM	73.07±1.72	69.88±3.12	76.74±1.20	76.14±1.09	73.52±2.07
	MUSA	79.20±0.65	83.22±2.35	74.57±3.92	87.08±0.51	81.09±0.29

**Table 11:** Comparison of frozen and trainable ROI-level 3D ViT encoder settings.

Encoder Setting	Initialization	Pretraining	Encoder Update	ACC (%)	AUC (%)	F1 (%)
MUSA	Random	None	Frozen	79.20±0.65	87.08±0.51	81.09±0.29
Random-Trainable	Random	None	Trainable	58.03±0.73	62.15±1.36	61.62±0.68

**Table 12:** Controlled analysis of input dimensionality and classifier capacity.

Model	Input Setting	ACC (%)	AUC (%)	F1 (%)
MUSA	Full 280,720-dimensional fused input	79.20	87.08	81.09
Parameter-matched control	Full-dimensional input with matched classifier capacity	75.34	83.19	77.03
1024-dimensional compressed-input baseline	Compressed to 1024 dimensions	71.60	79.31	73.42
Reduced-capacity MLP	Full 280,720-dimensional fused input with a smaller MLP	78.74	88.14	80.77

**Table 13:** Ranking results of the top 10 important brain-region pairs in the unified ROI semantic space.

Brain-Region Pair	Functional Domain	Pair Mean Weight	CV	L–R Difference
Postcentral	Sensorimotor	19.4224	0.1263	0.4897
Frontal_Sup	Frontoparietal control	19.2690	0.1242	0.2787
Occipital_Inf	Visual	19.2268	0.1243	0.6904
Frontal_Inf_Oper	Frontal control/language	19.2264	0.1302	0.6188
Precentral	Sensorimotor	19.1515	0.1267	0.1417
Temporal_Inf	Temporal association	19.1062	0.1294	0.3371
ParaHippocampal	Limbic memory	19.0444	0.1162	0.2516
Frontal_Mid_Orb	Orbitofrontal-limbic	19.0413	0.1245	1.5584
Temporal_Sup	Auditory/language	19.0130	0.1296	0.5172
Parietal_Inf	Parietal control	18.9903	0.1217	0.3459

Note: Mean weight denotes the average importance weight of each bilateral brain-region pair. CV denotes the coefficient of variation across folds. L–R difference denotes the absolute difference between the left and right homologous ROI weights. Brain-region pairs are sorted in descending order by mean weight.

## Data Availability

The data underlying this article were obtained from the REST-meta-MDD dataset of the Depression Imaging REsearch ConsorTium (DIRECT) and are available through the Psychological Science Data Bank at https://doi.org/10.57760/sciencedb.o00115.00013. Access to and use of the data are subject to the data-use procedures of the REST-meta-MDD Consortium, including completion of the Data Use Agreement and compliance with the relevant ethical requirements. No new participant-level neuroimaging data were collected in this study. The implementation code of MUSA and detailed reproduction instructions are publicly available at https://github.com/wanghonghua23/MUSA (accessed on 3 October 2026). The repository includes scripts for data splitting, FC construction, ROI-level 3D ViT feature extraction, multimodal feature combination, and classification, together with environment dependencies and data-organization instructions. Pre-extracted ROI-level 3D ViT features are also provided to facilitate reproduction of the classification experiments. The original MRI data are not redistributed in the MUSA repository. The preprocessing procedures used in this study are described in Section 3.2.

## Supplementary Materials

The following supporting information can be downloaded at https://www.mdpi.com/article/10.3390/s26206555/s1, Table S1: Subject-level paired statistical comparisons between MUSA and baseline models across single-feature conditions on the held-out test set (N = 476).

## Appendix A

**Algorithm A1** Unified-ROI MUSA for MDD/NCs Classification
**Require:** Raw multimodal data $D=\{\left(Y_{i}^{m},y_{i}\right)\}$, i=1,…,N, $y_{i}\in \left\{\mathrm{NCs},\mathrm{MDD}\right\}$ Precomputed FC matrices $C_{i}\in R^{R\times R}$ Selected feature combination M AAL atlas (R=116); random seed (42) **Ensure:** Predicted class probabilities ${\hat{p}}_{i}\in R^{2}$, ${\hat{y}}_{i}$ **Procedure: ROI Spatial Alignment and Voxel Extraction:** 1: **for** each imaging feature m∈M∖{FC} **do** 2: AAL atlas → sMRI grid/R-fMRI grid $\to L_{i}^{m}$ 3: $L_{i}^{m},Y_{i}^{m}\to$ ROI voxel sequence $x_{i,r}^{m}$ 4: $x_{i,r}^{m}\to$ near-cubic 3D block 5: Z-score normalization $\to X_{i,r}^{m}$ 6: **end for** **ROI Voxel Organization and 3D ViT-Based Feature Encoding:** 7: **For sMRI channels** {GM,WM,CSF}: $X_{i,r}^{m}\to$ sMRI 3D ViT-based ROI encoder $\to h_{i,r}^{m}\in R^{768}$ all ROI embeddings $\to H_{i}^{\mathrm{sMRI}}\in R^{R\times (3\times 768)}$ 8: **For R-fMRI channels**{ALFF,DC,ReHo,VMHC}: $X_{i,r}^{m}\to$ R-fMRI 3D ViT-based ROI encoder $\to h_{i,r}^{m}\in R^{768}$ all ROI embeddings $\to H_{i}^{\text{R-fMRI}}\in R^{R\times (4\times 768)}$ **Multimodal ROI Fusion:** 9: $H_{i}^{\mathrm{sMRI}},H_{i}^{\text{R-fMRI}}\to H_{i}^{\mathrm{img}}\in R^{R\times (7\times 768)}$ 10: Selected modality combination: $H_{i}^{\mathrm{img}}\to H_{i}^{\mathrm{sel}}\in R^{R\times (k\times 768)}$ 11: FC fingerprints: $C_{i}\to H_{i}^{\mathrm{FC}}\in R^{R\times R}$ 12: $F_{i}\leftarrow \mathrm{Concat}\left(H_{i}^{\mathrm{sel}},H_{i}^{\mathrm{FC}}\right)$ 13: $x_{i}\leftarrow \mathrm{vec}\left(F_{i}\right)$ **MLP Classification:** 14: D→ TrainVal/Test (8:2); TrainVal → 5-fold 15: **for** each fold k∈{1,…,5} **do** 16: [(a)] Fit StandardScaler on training subset only; transform $x_{i}\to x_{i}^{\prime }$ 17: [(b)] MLP([640,512,384,256]); Linear → BN → ReLU → Dropout AdamW$\left(\mathrm{lr}=9\times 10^{-4},\mathrm{wd}=1\times 10^{-4}\right)$; warmup(15) + cosine decay max epoch 400; patience 100 18: [(c)] best checkpoint (val loss) → Test $o_{i}\leftarrow \mathrm{MLP}\left(x_{i}^{\prime }\right)\in R^{2}$; ${\hat{p}}_{i}\leftarrow \mathrm{Softmax}\left(o_{i}\right)$ ${\hat{y}}_{i}\leftarrow \arg\max\left({\hat{p}}_{i}\right)$ 19: **end for** 20: **return** Fold-averaged ACC, SEN, SPE, AUC, F1 (mean ± std), predictions $\left({\hat{p}}_{i},{\hat{y}}_{i}\right)$
