# Supplementary material for: MUSA: A Multimodal MRI-Based Major Depressive Disorder Classification Model with a Unified ROI Semantic Space
Source: Sensors (Basel). 2026 Oct 15;26(20):6555. doi: 10.3390/s26206555 (PMC13708791; doi:10.3390/s26206555)
Supplement: Supplementary file 1 [file sensors-26-06555-s001.zip › sensors-4554639-supplementary.pdf]

Table S1. Subject-level paired statistical comparisons between MUSA and baseline models across single-feature conditions on the held-out test set (N = 476).

Feature	Comparison	ΔACC (pp)	95% CI for ΔACC (pp)	McNemar χ^2	Raw p	Holm-adjusted p
GM	MUSA vs ROI-Mean	+14.71	[8.17, 21.24]	18.70	< 0.001	< 0.001
GM	MUSA vs 3D DenseNet121	+4.20	[0.93, 7.47]	6.25	0.012	0.075
WM	MUSA vs ROI-Mean	+3.99	[0.54, 7.44]	5.08	0.024	0.097
WM	MUSA vs 3D DenseNet121	+7.56	[3.54, 11.58]	13.22	< 0.001	0.004
CSF	MUSA vs ROI-Mean	+2.31	[−0.56, 5.19]	2.47	0.116	0.232
CSF	MUSA vs 3D DenseNet121	+3.99	[0.40, 7.59]	4.69	0.030	0.097
ReHo	MUSA vs ROI-Mean	+5.04	[1.53, 8.56]	7.78	0.005	0.047
ReHo	MUSA vs 3D DenseNet121	+1.05	[−1.52, 3.62]	0.64	0.423	0.423
DC	MUSA vs ROI-Mean	+7.98	[3.65, 12.32]	12.67	< 0.001	0.004
DC	MUSA vs 3D DenseNet121	+4.62	[0.92, 8.33]	5.90	0.015	0.076
ALFF	MUSA vs ROI-Mean	+4.62	[1.15, 8.09]	6.72	0.010	0.067
ALFF	MUSA vs 3D DenseNet121	+4.83	[1.34, 8.32]	7.25	0.007	0.057
VMHC	MUSA vs ROI-Mean	+6.51	[2.50, 10.53]	9.91	0.002	0.016
VMHC	MUSA vs 3D DenseNet121	+6.72	[2.82, 10.63]	11.13	< 0.001	0.009
